# Laparoscopic management of pedicle torsion of adnexal cysts

**DOI:** 10.3892/ol.2013.1229

**Published:** 2013-03-05

**Authors:** YUXIA WANG, YUANYUAN XIE, XIAOQING WU, LU LI, YIFEI MA, XIAOYUAN WANG

**Affiliations:** Department of Obstetrics and Gynecology, Jinan Central Hospital, Shandong University, Jinan, Shandong 250013, P.R. China

**Keywords:** pedicle torsion of adnexal cyst, conservative surgery, laparoscopic surgery

## Abstract

Pedicle torsion of adnexal cysts results from the increased weight of cysts, longer length of the ovarian and suspensory ligaments or ovarian teratoma. Color doppler ultrasonography is particularly important for detecting suspected cyst torsion. Laparoscopy is becoming more important in the early diagnosis and treatment of adnexal cyst torsion due to its advantages, such as its minimally invasive nature, reduced acute stress reaction and facilitation of direct observation of intra-abdominal lesions. The present study analyzed 28 cases of laparoscopic torsion surgery. The laparoscopic conservative surgery rate was 75% and loss of endocrine function and fertility was avoided. Since the torsion duration is the only variable factor for avoiding oophorectomy, laparoscopic exploration should be performed as soon as possible when pedicle torsion of an adnexal cyst is suspected. Detorsion while retaining ovarian function did not increase the risk of thromboembolism and laparoscopic surgery was minimally invasive with faster recovery times and minimal impact on fertility. Furthermore, the study showed that the laparoscopic management of pedicle torsion of adnexal cysts is safe and reliable with the retention of ovarian endocrine and reproductive function.

## Introduction

Adnexal cyst torsion accounts for 3% of acute abdomen diseases in gynecology and includes pedicle torsion of ovarian and mesosalpinx cysts, which occur mainly in females between 20 and 39 years old. Pedicle torsion of adnexal cysts results from the increased weight of cysts, longer length of the ovarian and suspensory ligaments or ovarian teratoma. However, endometriosis of the ovary may not lead to torsion due to its invasive growth and adhesions. Pedicle torsion of the right side of the adnexal cyst commonly appears in the left side, due to the longer length of the right inherent ligament and relatively active bowel movements of the right terminal ileum and appendix. However, the sigmoid colon occupies the left side of the pelvis and torsion seldom occurs due to the narrow space. The clinical diagnosis of acute torsion includes symptoms of abdominal pain, nausea and vomiting. Gynecological examination and ultrasound are of great importance in mass detection. Color doppler ultrasonography is particularly useful in the detection of suspected cyst torsion. Ultrasound observation can usually detect pelvic abnormal echo-ray group, but cannot detect the ipsi-lateral ovary. The lack of a blood flow signal suggests arterial obstruction and the loss of ovarian activity (1,2). Radical surgery, such as direct oophorectomy, has previously been used in order to avoid the potential complication of venous thromboembolism (3–5). However, thromboembolism has not been reported particularly often. When pedicle torsion of an adnexal cyst is suspected, surgery should be performed as soon as possible. Laparoscopy is becoming more important in the early diagnosis and treatment of adnexal cyst torsion due to its advantages, such as its minimally invasive nature, reduced acute stress reaction and facilitation of direct observation of intra-abdominal lesions (6–8). The resetting of laparoscopic torsion makes ovary preservation possible, maintaining fertility. The present study analyzed 28 cases of laparoscopic torsion surgery.

## Materials and methods

### Study population

A total of 28 patients with confirmed pedicle torsion of adnexal cysts who underwent emergency laparoscopic surgery were recruited into the present study (Table I). Abdominal pain with nausea and vomiting appeared suddenly in the 28 patients following either changing position or intercourse. Until the emergency surgery, the pain lasted for between 3 and 18 h. The study was approved by the Ethics Committee of the Jinan Central Hospital, Shandong University, Jinan, China. Written informed consent was obtained from the patients.

### Technical methods

Laparoscopy was performed in all patients using three puncture sites. A veress needle was placed via a vertical incision into the navel ring and pneumoperitoneum was formed with a pressure of 12–15 mmHg to place a 10-mm trocar. Trocars (5-mm) were placed outside the lining of the navel and left and right iliac spine. The laparoscopic surgery involved three steps: diagnosis, ischemic lesion treatment and surgical treatment.

When pedicle torsion of an adnexal cyst was confirmed, blood circulation was observed and detorsion was slowly and gently performed using a non-traumatic vascular clamp. The cyst was punctured to aspirate cystic fluid prior to detorsion if it was large. The patients were divided into three groups according to the degree of torsion-induced ischemia and recovery speed: group A, relatively mild ischemia (ovaries and fallopian tubes appeared red in laparoscopy) and rapid recovery; group B, relatively severe ischemia (ovaries and fallopian tubes appeared dark red in laparoscopy) and recovery within 10 min; group C, ischemic necrosis and recovery time of >10 min. Conservative surgery and ovarian cyst or mesosalpinx cystectomy were performed for groups A and B, while group C underwent oophorectomy or salpingectomy when there was oviduct torsion.

### Statistical analysis

All variables were presented as the mean values ± standard error and P<0.05 was considered to indicate statistically significant differences. All statistical calculations were performed using SPSS 13.00.

## Results

### Technical results

Laparoscopic surgery succeeded without conversion to laparotomy or thromboembolism in all the 28 cases and the patients were discharged from hospital 3 days after surgery. There were 10 cases of mild ischemia (group A, 35.7%), 11 cases of severe ischemia (group B, 39.3%) and 7 cases of ischemic necrosis (group C, 25%). Conservative surgery and ovarian cyst or mesosalpinx cystectomy were performed in 21 cases (group A and B, 75%), while the remaining 7 cases underwent oophorectomy or salpingectomy (group C, 25%).

### Pain duration and torsion results

For the conservative surgery, patients experienced pain for an average of 5.4±1.4 h until they underwent emergency surgery, without exceeding 8 h. However, for those that underwent oophorectomy or salpingectomy, the pain lasted for an average of 12.8±3.1 h and all cases exceeded 8 h. There was a significant difference between the torsion results with regard to the pain duration, as is shown in Table II.

### Tumor type

During surgery, rapid pathological examinations were performed in 15 out of the 28 cases. The 28 postoperative pathology reports all showed benign tumors, with 15 cases of ovarian mature cystic teratomas, 6 ovarian serous cystadenomas, 2 ovarian mucinous cystadenomas, 3 ovarian crown cysts and 2 mesosalpinx cysts.

### Follow-up

The 21 patients who underwent conservative surgery were followed up for between 3 and 12 months. The ipsilateral ovary size, follicular development and ovary blood flow appeared normal in vaginal or abdominal color doppler ultrasound in all cases 3 months after surgery. The 8 patients who wanted to have children all conceived naturally within 3-10 months with full term delivery.

## Discussion

For pedicle torsion, the torsion or pain duration, from the appearance of abdominal pain to the diagnosis and surgical treatment, determines whether the ovary is retained. In the present study, conservative surgery was performed in cases with a pain duration <8 h, avoiding the loss of endocrine function and reproductive fertility. By contrast, ovaries or fallopian tubes were removed due to irreversible ischemic necrosis in cases where the pain duration was >8 h, consistent with previous studies (9). However, it has been shown in a previous study that torsion duration has no effect on the technical results (10). It was previously considered that thromboembolism due to detorsion would result in complications such as pulmonary embolism, so radical surgery and ipsilateral oophorectomy are the recommended treatments (11). However, it has been reported that the incidence of pulmonary embolism is only 0.2% and it is not increased following detorsion (12). There were no cases of thromboembolism in the present study.

When torsion has been confirmed by laparoscopy, the degree of ischemia should be examined and reset using a non-traumatic vascular clamp. If the cyst is too large to reset, it may be punctured to aspirate cystic fluid prior to detorsion. The color and speed of circulation recovery of the ovary and fallopian tube should be observed. For mild ischemia, cystectomy may be performed. For severe ischemia, when the color is dark, cysts may be punctured to decrease the tension and soaked in saline for 10 min to observe whether the blood circulation recovers. Conservative surgery remains feasible if circulation recovers within 10 min. If not, oophorectomy or salpingectomy are recommended.

In the present study, the laparoscopic conservative surgery rate was 75% and the loss of endocrine function and fertility was avoided. Since the torsion duration is the only variable factor for preventing oophorectomy, laparoscopic exploration should be performed as soon as possible when pedicle torsion of an adnexal cyst is suspected. It has been shown that detorsion while retaining ovarian function does not increase the risk of thromboembolism and laparoscopic surgery is minimally invasive with faster recovery times and minimal impact on fertility. Furthermore, the study showed that the laparoscopic management of pedicle torsion of adnexal cysts is safe and reliable with the retention of ovarian endocrine and reproductive function.

## Figures and Tables

**Table I:** Patient characteristics.

Characteristic	Value
Age range, years (median)	18-39 (25.6)
Married, n	20
Unmarried, n	8
Nullipara, n	16
No history of abdominal surgery, n	28
Gynecological or rectal examination, n	
Palpable pelvic mass	24
Unpalpable	4
Ultrasound examination, n	
Adnexal cyst or solid mass with diameter 5–8 cm	28
Total, n	28

**Table II:** Association of abdominal pain duration with torsion surgery selection.

Surgery selected	No. of cases	Average pain duration (h)
Conservative surgery	21	5.4±1.4
Oophorectomy or salpingectomy	7	12.8±3.1^a^

a Significantly different compared with the conservative surgery cases (P<0.05).
